# Circuitous Path to Live Donor Liver Transplantation from the Coordinator’s Perspective

**DOI:** 10.3390/jpm11111173

**Published:** 2021-11-10

**Authors:** Hui-Ying Lin, Cheng-Maw Ho, Pei-Yin Hsieh, Min-Heuy Lin, Yao-Ming Wu, Ming-Chih Ho, Po-Huang Lee, Rey-Heng Hu

**Affiliations:** 1 Department of Nursing, National Taiwan University Hospital and College of Medicine, Taipei 100, Taiwan; fifi3123@gmail.com (H.-Y.L.); Peiyin3111@ntuh.gov.tw (P.-Y.H.); mhlin@ntuh.gov.tw (M.-H.L.); 2 Department of Surgery, National Taiwan University Hospital and College of Medicine, Taipei 100, Taiwan; wyaoming@gmail.com (Y.-M.W.); mcho1115@ntu.edu.tw (M.-C.H.); pohuang1115@ntu.edu.tw (P.-H.L.); rhhu@ntu.edu.tw (R.-H.H.); 3 Department of Surgery, E-Da Hospital, I-Shou University, Kaohsiung 824, Taiwan

**Keywords:** live donor liver transplantation, intend to donate, root cause, risk factor

## Abstract

**Background:** The live donor liver transplantation (LDLT) process is circuitous and requires a considerable amount of coordination and matching in multiple aspects that the literature does not completely address. From the coordinators’ perspective, we systematically analyzed the time and risk factors associated with interruptions in the LDLT process. **Methods:** In this retrospective single center study, we reviewed the medical records of wait-listed hospitalized patients and potential live donors who arrived for evaluation. We analyzed several characteristics of transplant candidates, including landmark time points of accompanied live donation evaluation processes, time of eventual LDLT, and root causes of not implementing LDLT. **Results:** From January 2014 to January 2021, 417 patients (342 adults and 75 pediatric patients) were enrolled, of which 331 (79.4%) patients completed the live donor evaluation process, and 205 (49.2%) received LDLT. The median time from being wait-listed to the appearance of a potential live donor was 19.0 (interquartile range 4.0–58.0) days, and the median time from the appearance of the donor to an LDLT or a deceased donor liver transplantation was 68.0 (28.0–188.0) days. The 1-year mortality rate for patients on the waiting list was 34.3%. Presence of hepatitis B virus, encephalopathy, and hypertension as well as increased total bilirubin were risk factors associated with not implementing LDLT, and biliary atresia was a positive predictor. The primary barriers to LDLT were a patient’s critical illness, donor’s physical conditions, motivation for live donation, and stable condition while on the waiting list. **Conclusions:** Transplant candidates with potential live liver donors do not necessarily receive LDLT. The process requires time, and the most common reason for LDLT failure was critical diseases. Aggressive medical support and tailored management policies for these transplantable patients might help reduce their loss during the process.

## 1. Introduction

Live donor liver transplantation (LDLT) is the primary treatment option for end-stage liver disease in countries that have organ shortages [1,2]. LDLT also provides a timely rescue for transplant candidates who may not survive long enough to receive liver transplants from deceased donors [3] However, live liver donation surgeries are not without risks and complications [4,5] Therefore, much effort has been devoted to expanding the maximal operability of live liver donations [6] and to reducing complications as much as is feasible.

In addition to meticulous surgery for live donors and recipients [6], careful planning and evaluation of the LDLT process are essential. Although the evaluation process may differ between centers, the percentages of “disqualified” donors in reports after 2010 have ranged from 48.5% to 69.7% [7,8,9,10] Common donor-related reasons for not proceeding with LDLT were reluctance [7,8,10], fatty liver [8,9,10], small remnant liver volume [8,9,10], anatomical variation [8], and medical problems [7]. Wait-list death is a highly common recipient candidate-related reason [8], and recipient candidate characteristics associated with donor acceptance are a younger age, lower Model for End-stage Liver Disease (MELD) score, and shorter time from listing to the first donor evaluation (cutoff value: 23 days) [11]. The LDLT process is circuitous and requires much coordination and matching of multiple aspects. However, the LDLT process, risk factors, and root-cause analysis are not fully addressed in the literature. We systematically analyzed the path and risk factors associated with interruption during the LDLT process from the perspective of LDLT coordinators.

## 2. Methods

The Institutional Review Board (IRB) of National Taiwan University Hospital (NTUH), Taipei, Taiwan, approved this study (NTUH REC: 201701044RIND and 202004053RINB). Because this was a retrospective study using chart review, the IRB waived the need for informed consent.

## 3. Patients

We retrospectively reviewed the medical records of hospitalized patients who were evaluated for liver transplants and wait-listed on the Taiwan Organ Registry and Sharing Center from January 2014 to January 2021. Candidates whose potential live donors appeared for donation evaluation were included. The index date was the date of approval for pre-claim review (wait-listing). Patients rejected during pre-claim review or without potential live donors were excluded.

## 4. Live Donor Evaluation Process for LDLT

According to Taiwan Human Organ Transplant Act, live donor candidates should be aged ≥18 years and be a fifth degree relative of the transplant candidates [12]. When patients were evaluated for liver transplantation, medical team informed patients and their family members about the option of LDLT. Medical team would hold a family meeting, if requested, for more detailed information of LDLT. When potential live donors expressed their interest in live liver donation, they were referred to transplant clinics for further evaluation. The evaluation process included first stage outpatient (blood type identification, health check, and presence of hepatitis virus) and second stage inpatient (image evaluation and psychosocial interview) management. Potential live donors were assessed and interviewed alone for autonomy and motivation by psychiatrists and social workers. Regular multidisciplinary transplant meetings, attended by hepatologists (including pediatricians), liver transplant surgeons, radiologists, and social workers, were held to review and discuss the feasibility of LDLT for each pair. LDLT was arranged and performed after receiving the approval from the transplant meeting members and the clinical ethics committee of NTUH. To foster the LDLT process, since July 2015 the approval decision of living unrelated liver donation or live donors with age less than 20 has been delegated to the clinical ethics committees of the hospitals and is no longer made from central government. Moreover, from April 2019, copayment of live liver donation was exempted from National Health Insurance.

## 5. Demographic Parameters

Demographic information, namely sex, age, height, weight, underlying liver diseases and comorbidities (presence of hepatitis B virus (HBV), hepatitis C virus (HCV), alcohol use, hepatocellular carcinoma (HCC), biliary atresia (BA), diabetes mellitus (DM), and hypertension), MELD [13] or Pediatric End-Stage Liver Disease (PELD) [14] scores, and clinical variables at the time of the pre-claim review, were collected. Data were collected on the evaluation progress of live liver donors, including the dates the donors arrived and the reasons for ultimately not completing LDLT.

## 6. Criteria for Waiting List Exclusion

Transplant candidates were excluded from the waiting list when contraindications emerged or the experts deemed the prospect of LDLT as futile. For example, candidates with distant metastases of HCC or profound septic shock were excluded. Transplant panel specialists retained the final decision for waiting list exclusion.

## 7. Outcome Measurements

The patients were followed-up until their death or August 2021. The event date was either the date of death, liver transplantation, LDLT, or last follow-up. The date of potential live donor appearance was deemed the same date as that of pre-claim approval if the date of the live donor evaluation was earlier than the start date of wait-listing. The primary outcome was implications of LDLT. Secondary outcomes were overall survival and survival for patients on the waiting list.

## 8. Root-Cause Analysis

Original medical records for every patient included in the study were jointly analyzed by a senior transplant coordinator (LHY) with a transplant surgeon (HCM). Data were subdivided into three broad categories of barriers: eligible transplant candidates, eligible donors, and matching and pairing. After data consistency and accuracy were reviewed on a case-by-case basis, a synopsis was written and anonymously presented to independent senior transplant surgeons qualified as experts. After evaluating each patient’s data, the expert panel identified the primary barriers to successful live liver donation and categorized transplant candidates using a fishbone diagram.

## 9. Statistical Analysis

Descriptive statistics are expressed as a mean ± standard deviation or number (percentage) where appropriate. The Student’s *t* test, χ^2^ test, or Fisher’s exact test was used, where appropriate, to compare the variables. Cumulative survival rates, probabilities of donor appearance, and probabilities of successful LDLT were estimated using the Kaplan–Meier method and compared using the log-rank test and post hoc analysis. Logistic regression modelling was employed for univariable and multivariable analyses. Statistical significance was indicated by a two-sided *p* value of <0.05. Analyses were performed using SPSS version 21.0 (IBM Corporation, Armonk, NY, USA).

## 10. Results

### 10.1. Demographics

**Figure 1:**
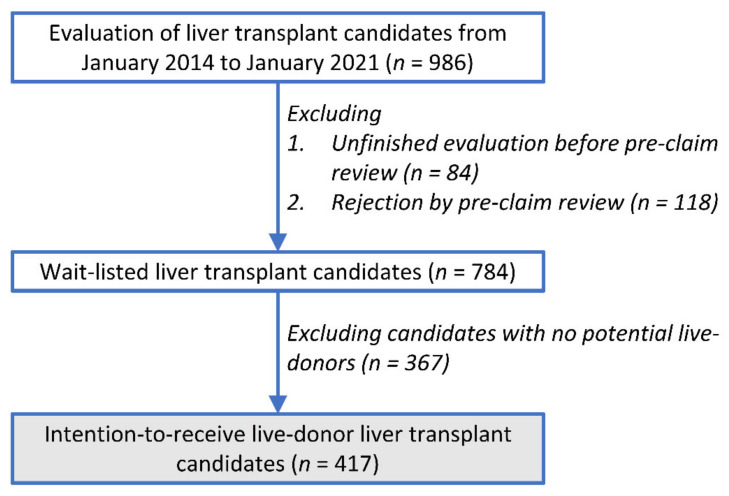
Patient flow diagram.

During the study period, 968 patients were evaluated for liver transplantation and 784 were wait-listed (Figure 1). After 367 patients without potential live liver donors were excluded, 417 (53.2%, 417/784) patients were included in the study (Figure 1). This cohort included 342 adults and 75 pediatric patients.

In total, 331 candidates (260 adults and 71 children) had at least one potential live donor who completed the second stage of donor evaluation. Table 1 displays the patient characteristics. In the adult subgroup, most patients were men, carriers of HBV, and had esophageal varices (EVs) and ascites; the average age was 55.3 ± 9.8 years (Table 1A). In the pediatric group, half of the patients were male or had underlying BA, and the average age was 3.1 ± 4.2 years (Table 1B). In total, 153 adults and 52 pediatric transplant candidates received LDLT, and 19 adult and 2 pediatric patients received deceased donor liver transplants (DDLTs). In adults, compared with the group who successfully received LDLT, other candidates had more causes of HBV; higher presence of hypertension and encephalopathy, MELD scores, and serum levels of total bilirubin; fewer causes of HCV; and a smaller presence of HCC (borderline significance; Table 1A). Higher weights and less BA etiology and EVs (borderline significance) were observed (Table 1B) in pediatric counterpart. In adults, compared with patients who died without having received transplants, living patients without transplants had lower MELD scores, serum levels of total bilirubin, and international normalized ratios; less presence of encephalopathy; and higher serum albumin levels (Table 1A). Pediatric patients exhibited lower PELD score and serum levels of total bilirubin (borderline significance) and fewer ascites (Table 1B).

### 10.2. Survival and Mortality of Patients on the Waiting List

**Figure 2:**
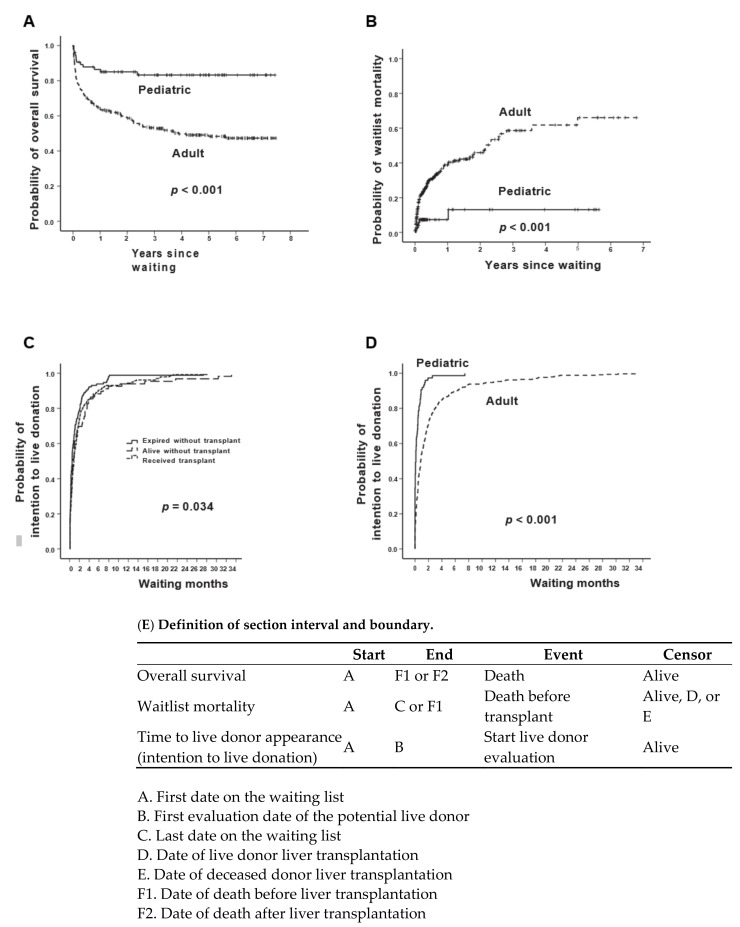
Probability curves of candidates with “intend to donate” live donors. Overall survival (**A**) and mortality rates for patients on the waiting list (**B**) in adult and pediatric subgroups. Occurrence of potential live liver donors in 3 subgroups (died, alive without transplant, and received transplant) (**C**) and in adult and pediatric subgroups (**D**). (**E**) Definition of section interval and boundary.

The 1-, 3-, 5-, 7-, and 9-year overall survival rates after placement on the waiting list were 68.2%, 63.4%, 58.2%, 55.7%, and 54.6%, respectively, among the “intend for LDLT” candidates. Overall survival in the pediatric subgroup was superior to that in the adult subgroup (*p* < 0.001; Figure 2A). For candidates who eventually received liver transplants, the 1-, 3-, 5-, 7-, and 9-year overall survival rates after placement on the waiting list were 89.1%, 84.7%, 80.4%, 76.9%, and 76.1%, respectively. The 1-, 3-, 5-, 7-, and 9-year mortality rates of wait-listed patients were 34.3%, 41.2%, 52.1%, 54.4%, and 57.4%, respectively, among the “intention for live donation” candidates. The mortality rate for those on the waiting list was higher in the adult subgroup than in the pediatric subgroup (*p* < 0.001; Figure 2B).

### 10.3. Probability of Intention for Live Donation and Time Course of Patients on the Waiting List with Live Donors’ Initial Intention to Donate

The median time from being wait-listed to potential live donor appearance was 19.0 (interquartile range, IQR 4.0–58.0) days. The probability of intention for liver donation differed for the subgroups who died without having received a transplant, lived without a transplant, and received a transplant (*p* = 0.034), with post hoc significance in comparison subgroups (died vs. alive, *p* = 0.026; died vs. transplanted, *p* = 0.015; Figure 2C). Moreover, the pediatric subgroup exhibited a higher probability of intention for live donation than the adult subgroup did (*p* < 0.001; Figure 2D).

The median time from live donor appearance to either LDLT or DDLT was 68.0 (IQR 28.0–188.0) days. Most (90.2%) of the candidates in this study received LDLT within 138 days of the initiation of live donor evaluation.

Among the 205 patients who eventually received LDLT, the median time from placement on the waiting list to live donor appearance, time from live donor appearance to LDLT, and total waiting time were 65.0 (IQR, 34.0–94.5), 24.0 (7.0–61.0), and 101.0 (58.5–170.0) days, respectively. Among the 124 patients who died without having received a transplant, the median time from being placed on the waiting list to live donor appearance, time from live donor appearance to death, and total waiting time were 13.0 (IQR, 3.0–47.8), 29.5 (10.5–89.8), and 44.5 (19.3–167.0) days, respectively. The differences of the three duration periods between the LDLT group and the group who died without having received a transplant were significant, with *p* = 0.011, *p* < 0.001, *p* < 0.001, respectively.

Thirty-five live donors with intention to donate began evaluation before the pre-claim approval as transplant candidates. Among the 35 transplant candidates, 19 received LDLT, one received DDLT, eight died before transplant, five were waiting in a stable condition, one recovered, and one was lost to follow-up after 15 months.

### 10.4. Factors Associated with Successful LDLT

According to a univariate analysis, older age, adult patients, HBV carriers, high MELD or PELD scores (≥30), presence of hypertension or encephalopathy, and increased total bilirubin level were significantly associated with successful LDLT (Table 2). BA was significantly associated with successful LDLT (Table 2). According to a multivariable analysis, factors remaining significant were whether the patient was an HBV carrier, the presence of hypertension, encephalopathy, or BA (positive association), and an increased total bilirubin level (negative; Table 2).

### 10.5. Root-Cause Analysis

**Figure 3:**
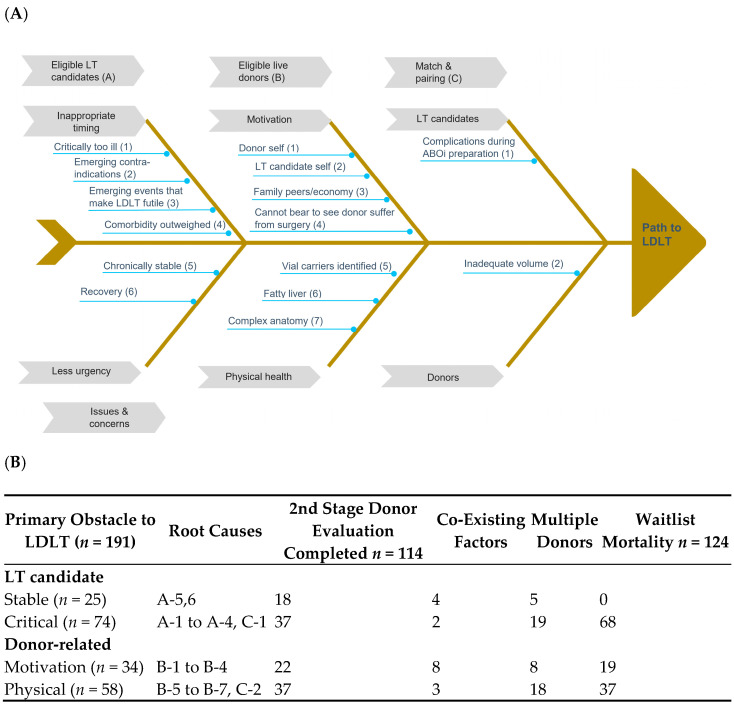
Root-cause analysis of failed live liver donations. (**A**). Fishbone diagram illustrating potential barriers to preemptive LDLT. (**B**). Category summary. LDLT, live donor liver transplant; LT, liver transplant.

Documented barriers to a successful LDLT process in transplant candidates with “intend to donate” live donors are illustrated in Figure 3A. Major challenges for eligible transplant candidates included inappropriate timing (being too ill with profound septic shock for example), emerging contraindications (cancer metastases) or events (myocardial infarction or intracerebral hemorrhage), and having less urgent need. Major challenges for eligible live donors included motivation (either donor self or family peers) and health (fatty liver, previously unidentified diseases, or complex anatomical variations). Obstacles to matching and pairing were mainly caused by insufficient donated or remaining liver volumes after pairing and emerging events during preparation of ABO (blood group)-incompatible LDLT (sepsis under prophylactic immunosuppression).

In total, 21 patients who received DDLT were not included. Three patients (two died without having received a transplant) with unfinished potential live donor evaluations for unidentified causes were not included.

Figure 3B displays the effects of major impediments to the LDLT process and waiting list mortality. The hierarchy of primary factor determination was “critical transplant candidate”, “motivation”, “donor health and anatomy”, and “stable transplant candidate”. Among 191 candidates with “intend to donate” live liver donors, the primary barrier was “critical LT candidate”, and other barriers included “physical (donor)”, “motivation”, and “stable LT candidate”. Fifty transplant candidates (50/191, 26.2%) had multiple “intend to donate” donors. In total, 114 (60.6%) candidates had at least one potential donor who completed the second stage evaluation, and their waiting list mortality rate was 64.9% (124/191). More than half (68) of the patients who died were categorized as “critical”, followed by “donor physical” (37). The median time from being placed on the waiting list to donor appearance and from donor evaluation to waiting list exclusion for transplant candidates categorized as “critical” were 13 and 15.5 days, respectively. Thirty-four transplant candidates had “live donor motivation”, but more than half (19/34) died before receiving a transplant. Other coexisting factors were noted among 8.9% (17/191) of the transplant candidates. Notably, five of the nine transplant candidates preparing for ABO-incompatible LDLT died during the process. Among 58 cases in which the donor’s physical condition was the primary barrier, 28 were fatty liver-related, nine donors had an insufficient remnant liver, and 37 transplant candidates out of 58 died without having received a transplant.

## 11. Discussion

This study revealed four major findings. First, more than half (417/784, 53.2%) of transplant candidates had at least one potential “intend to donate” live liver donor. Most (331/417, 79.4%) finished the donor evaluation process, and nearly half (205/417, 49.2%) received LDLT. Second, underlying liver diseases (HBV and BA), liver disease severity (total bilirubin and encephalopathy), and hypertension were associated with receiving LDLT. Among them, BA was the only positive predictor. Third, the median time from being wait-listed to the appearance of a potential live donor was 19.0 (IQR 4.0–58.0) days, and that from live donor appearance to either LDLT or DDLT was 68.0 (IQR 28.0–188.0) days. Finally, the primary barriers to LDLT were, in decreasing frequency, critical transplant candidates, donor physical conditions, motivation of live donation, and stable transplant candidates.

Our study indicates that a critical or inappropriate status of transplant candidates was the primary reason for exclusion from the waiting list. This is consistent with a report by Pamecha et al. [8] Trotter et al. [11] suggested that high MELD scores threatened the success of receiving LDLT. In our study, high MELD or PELD scores (≥30) did not favor LDLT in the univariable analysis, but the effect was not observed (and even reversed) in the multivariable analysis. The probable reasons for critical patient as a barrier to LDLT could be the candidate was too sick and passed away before transplant surgery, or the potential live donors or the medical team were hesitant to push live donation as outcomes in recipients were thought to be poorer. The existence of stable candidates in our cohort and other factors (e.g., BA) included in the adjustment may have altered the statistics. Despite potential live donors being available, LDLT was not guaranteed for children with non-BA conditions. While most of these pediatric transplant candidates had metabolic liver diseases which could be managed conservatively, family and pediatric medical team would try medical and diet control as the alternative. On the contrary, medical condition in children with BA progressed more rapidly which urged family and pediatric team members to proceed to LDLT in a timely manner. In our study, presence of HBV was another risk factor associated with not implementing LDLT. Vertical transmission from asymptomatic carrier mothers to their offspring explains most HBV infection in Taiwan [15], which means a large group of relatives, who could be potential live donor pools, were HBV carriers and not ideal for liver donation. Nevertheless, the belief that the presence of live donors eliminates the necessity to provide optimal patient care is dangerous. Moreover, whether underlying biological plausibility or associated unknown factors can explain the presence of hypertension as another barrier to LDLT is unknown and warrants further external validation.

Physical condition and motivation were two major donor-related factors in our study. As advancements in imaging and surgical science are made, the barriers of insufficient graft volume (to maintain enough remnant liver volume) or anatomical variation may have less of an effect [16,17,18,19]. The increasingly crucial matter of a donor’s fatty liver reflects the global obesity epidemic, and future therapeutic developments may lead to manageable solutions [20,21]. Negative motivations may result from the donors, significant others, or family economic concerns. Although the concern that LDLT was more expensive than DDLT had decreased for the copayment exemptions of live donation since April 2019, donors may suffer professionally due to loss of workdays and salary. The exact impact of financial implications on our findings was inaccessible. Reimbursing live organ donors for incurred nonmedical expenses may alleviate the impedance to LDLT [22,23,24].

A limitation of this study is that not all of our results may be externally applicable because of the differences in government regulations, sociocultural backgrounds, institutional policies, and operations management. However, we illustrated the difficulty of the LDLT process, addressed the risk factors, and analyzed the root causes of LDLT. Some of our results are consistent with those of previous studies.

In conclusion, half of transplant candidates with “intend to donate” live donors failed to receive LDLT. The most common reason for excluding patients from the waiting list was the existence of critical diseases. The median time between placement on the waiting list and the appearance of the donor was 19 days, and the preparation time for the transplant was 68 days. Therefore, additional time is required, and LDLT is not guaranteed for all patients, especially adults with HBV and children without BA. Aggressive medical care is essential for transplantable patients. The current data may serve as a guide for liver allocation policy makers.

## Figures and Tables

**Table 1:** Characteristics of intention-to-receive live donor transplant candidates. Live donor transplants vs. others in adult (**A**) and pediatric (**B**) patients.

**(A)**							
**Adult**	**All** ***n* = 342**	**Live liver Donation** ***n* = 153**	**Others** ***n* = 189**				
				***p***	Alive without Transplant * ***n* = 52**	**Expired** ***n* = 118**	***p***
Age	55.3 (9.8)	55.2 (9.3)	55.3 (10.3)	0.883	54.9 (10.5)	56.0 (10.4)	0.521
Male gender	237 (69.3)	105 (68.6)	132 (69.8)	0.815	35 (67)	81 (68.6)	0.860
O blood type	144 (42.1)	60 (39.2)	84 (44.4)	0.322	26 (50)	51 (43.2)	0.504
Height (cm)	164.4 (0.6)	164.3 (9.1)	164.4 (12.1)	0.932	164.2 (8.7)	163.7 (13.8)	0.811
Body weight (kg)	68.7 (15.2)	67.4 (14.5)	69.8 (15.8)	0.155	66.5 (14.6)	70.3 (16.8)	0.150
Underlying liver disease							
HBV	194 (56.7)	74 (48.4)	120 (63.5)	0.006	29 (56)	77 (65.3)	0.303
HCV	65 (19.0)	37 (24.2)	28 (14.8)	0.037	9 (17)	19 (16.1)	0.826
Alcohol	49 (14.3)	20 (13.1)	29 (15.3)	0.642	10 (19)	16 (13.6)	0.361
HCC	104 (30.4)	55 (35.9)	49 (25.9)	0.058	13 (25)	32 (27.1)	0.852
Extra-hepatic							
DM	87 (25.4)	39 (25.5)	48 (25.4)	>0.999	15 (29)	28 (23.7)	0.566
Hypertension	72 (21.1)	19 (12.4)	53 (28.0)	0.001	17 (33)	32 (27.1)	0.468
MELD	20.0 (10.7)	17.0 (9.7)	22.5 (10.8)	<0.001	16.4 (8.3)	25.3 (10.7)	<0.001
Total bilirubin (mg/dL)	12.1 (13.8)	8.2 (11.4)	15.3 (14.9)	<0.001	9.6 (12.6)	18.0 (15.2)	<0.001
INR	2.5 (9.5)	3.3 (14.2)	1.9 (1.2)	0.250	1.5 (0.7)	2.1 (1.3)	0.001
Creatinine (mg/dL)	1.3 (1.2)	1.2 (1.3)	1.3 (1.1)	0.259	0.9 (0.4)	1.5 (1.2)	<0.001
Sodium (mEq/L)	133.0 (18.2)	133.3 (16.8)	132.9 (19.2)	0.880	129.2 (30.6)	134.6 (7.9)	0.292
Albumin (g/dL)	3.1 (0.6)	3.1 (0.5)	3.0 (0.7)	0.422	3.3 (0.8)	2.9 (0.5)	0.004
EV	195 (57.0)	94 (61.4)	101 (53.4)	0.154	31 (60)	61 (51.7)	0.404
Encephalopathy	141 (41.2)	40 (26.1)	101 (53.4)	<0.001	11 (21)	78 (66.1)	<0.001
Ascites	206 (60.2)	91 (59.5)	115 (60.8)	0.825	27 (52)	75 (63.6)	0.176
**(B)**							
**Pediatric**	**All** ***n* = 75**	**Live liver Donation** ***n* = 52**	**Others** ***n* = 23**				
				***p***	Alive without Transplant * ***n* = 15**	**Expired** ***n* = 6**	***p***
Age (years)	3.1 (4.2)	2.8 (3.8)	3.9 (5.1)	0.355	4.8 (5.6)	2.6 (4.0)	0.405
Male gender (%)	39 (52)	27 (52)	12 (52)	>0.999	7 (47)	3 (50)	>0.999
O blood type (%)	33 (44)	25 (48)	9 (39)	0.616	8 (53)	1 (17)	0.178
Height (cm)	89.2 (68.3)	90.7 (77.0)	85.5 (42.2)	0.763	91.4 (48.2)	77.8 (33.2)	0.540
Body weight (kg)	18.4 (11.2)	12.5 (10.5)	21.1 (19.4)	0.049	25.6 (21.0)	15.3 (16.0)	0.298
Underlying liver disease							
Biliary atresia	37 (49)	31 (60)	6 (26)	0.012	2 (13)	2 (33)	0.544
PELD	18.4 (11.2)	17.6 (10.3)	20.3 (13.4)	0.375	17.1 (10.1)	30.8 (18.4)	0.063
Total bilirubin (mg/dL)	15.2 (11.6)	14.6 (10.4)	16.6 (14.1)	0.526	12.9 (11.4)	26.3 (17.4)	0.064
INR	1.6 (2.0)	1.3 (0.6)	2.3 (3.5)	0.228	2.6 (4.3)	2.0 (1.1)	0.770
Creatinine (mg/dL)	0.4 (0.7)	0.5 (0.8)	0.3 (0.2)	0.388	0.4 (0.2)	0.3 (0.1)	0.230
Sodium (mEq/L)	134.4 (2.8)	134.2 (2.9)	134.8 (2.2)	0.630	135.7 (2.2)	134.0 (1.4)	0.389
Albumin (g/dL)	3.5 (0.6)	3.5 (0.6)	3.5 (0.6)	0.869	3.6 (0.5)	3.5 (0.7)	0.945
EV (%)	29 (39)	24 (46)	5 (22)	0.071	3 (20.0)	2 (33)	0.598
Encephalopathy (%)	20 (27)	12 (23)	8 (35)	0.396	5 (33)	3 (50)	0.631
Ascites (%)	29 (39)	21 (40)	8 (35)	0.798	3 (20)	5 (83)	0.014

* (in A) Patients who received deceased donor liver transplant (*n* = 19) were excluded; * (in B) deceased donor liver transplant, *n* = 2.HBV, hepatitis B virus; HCV, hepatitis C virus; HCC, hepatocellular carcinoma; DM, diabetes mellitus; MELD, Model for End-stage Liver Disease; INR, international normalized ratio; EV, esophageal varices; PELD, Pediatric End-Stage Liver Disease.

**Table 2:** Factors associated with successful live liver donation in univariable and multivariable analyses. Patients who received deceased donor liver transplant were excluded.

	Univariable			Multivariable		
	OR	95 CI	*p*	OR	95 CI	*p*
Age	0.98	0.97–0.99	<0.001	0.99	0.96–1.02	0.380
Adult patients	0.36	0.21–0.63	<0.001	1.97	0.27–14.61	0.507
Height	0.99	0.99–1.00	0.022	1.01	0.99–1.02	0.438
Weight	0.98	0.98–0.99	<0.001	0.99	0.97–1.01	0.238
Male gender	0.89	0.59–1.35	0.595			
Donor show up time	1.01	1.00–1.00	0.329			
High MELD or PELD score (≥30)	0.41	0.24–0.70	0.001	1.15	0.55–2.41	0.708
Hypertension	0.33	0.19–0.58	<0.001	0.41	0.22–0.77	0.006
Underlying liver disease						
HBV	0.45	0.30–0.68	<0.001	0.55	0.33–0.93	0.026
HCV	1.28	0.75–2.19	0.364			
HCC	1.19	0.75–1.86	0.462			
Alcohol	0.69	0.37–1.28	0.233			
Biliary atresia	8.33	2.88–24.08	<0.001	4.89	1.29–18.51	0.020
EV	1.31	0.88–1.95	0.177			
Ascites	0.89	0.60–1.32	0.554			
Encephalopathy	0.33	0.22–0.50	<0.001	0.47	0.28–0.79	0.004
Total bilirubin	0.97	0.95–0.98	<0.001	0.97	0.95–0.99	0.013
INR	1.01	0.98–1.04	0.416			
Creatinine	0.85	0.71–1.04	0.109			

OR, odds ratio.

## Data Availability

The datasets used and analyzed during the current study are available from the corresponding author upon reasonable request.

## Abbreviations

BA	biliary atresia
CI	confidence interval
DDLT	deceased donor liver transplantation
DM	diabetes mellitus
EV	esophageal varices
HBV	hepatitis B virus
HCC	hepatocellular carcinoma
HCV	hepatitis C virus
INR,	international normalized ratio
IQR	interquartile range
IRB	Institutional Review Board
LDLT	live donor liver transplantation
LT	liver transplant
MELD	Model for End-stage Liver Disease
OR	odds ratio
PELD	Pediatric End-Stage Liver Disease
